# Supplementary material for: How accurately can one predict drug binding modes using AlphaFold models?
Source: eLife. 2023 Dec 22;12:RP89386. doi: 10.7554/eLife.89386 (PMC10746139; doi:10.7554/eLife.89386)
Supplement: Supplementary file 1. [file elife-89386-supp1.docx]

| *Supplementary Table 1. Structures in benchmark* | | | |
| --- | --- | --- | --- |
| Uniprot ID | Protein name | Class | PDB structure codes |
| AGRG3 | Adhesion G Protein-Coupled Receptor G3 | B | 7D76,7D77 |
| NK1R | Neurokinin-1 Receptor | A | 6E59,6HLL,6HLP,6J20 |
| 5HT2A | 5-Hydroxytryptamine Receptor 2A | A | 6A93,6A94,6WGT,6WH4,6WHA |
| ADA2A | Alpha-2A adrenergic receptor | A | 6KUX,6KUY |
| CNR2 | Cannabinoid Receptor 2 | A | 5ZTY,6KPF,6PT0 |
| DRD1 | Dopamine Receptor D1 | A | 7CKW,7CKX,7CKY ,7CRH, 7JOZ,7JVP,7JVQ,7LJD |
| CLTR2 | Cysteinyl Leukotriene Receptor 2 | A | 6RZ6,6RZ7,6RZ8,6RZ9 |
| CLTR1 | Cysteinyl Leukotriene Receptor 1 | A | 6RZ4,6RZ5 |
| MTR1A | Melatonin Receptor 1A | A | 6ME2,6ME4,6ME5,6PS8 |
| MTR1B | Melatonin Receptor 1B | A | 6ME7,6ME9 |
| PTAFR | Platelet Activating Factor Receptor | A | 5ZKP,5ZKQ |
| TA2R | Thromboxane A2 Receptor | A | 6IIU,6IIV |
| PE2R3 | Prostaglandin E2 Receptor EP2 Subtype | A | 6AK3,6M9T |
| PE2R4 | Prostaglandin E2 Receptor EP4 Subtype | A | 5YHL,5YWY,7D7M |
| PD2R2 | Prostaglandin D2 Receptor 2 | A | 6D26, 6D27 |
| 5HT1A | 5-Hydroxytryptamine Receptor 1A | A | 7E2Y,7E2Z |
| GPBAR | G protein-coupled bile acid receptor 1 | A | 7CFM,7CFN |
| PE2R2 | Prostaglandin E2 Receptor EP2 Subtype | A | 7CX2,7CX3,7CX4 |
